# Meis1 Regulates Nociceptor Development and Behavioral Response to Tactile Stimuli

**DOI:** 10.3389/fnmol.2022.901466

**Published:** 2022-07-06

**Authors:** Zheng Cao, Chengcheng Huang, Fumin Lu, Xuequan Jiang, Yong Hu, Cheng Cao, Zijing Liu

**Affiliations:** ^1^Beijing Institute of Biotechnology, Beijing, China; ^2^School of Biological Engineering and Food Science, Hubei University of Technology, Wuhan, China; ^3^General Hospital of Central Theater Command, Wuhan, China

**Keywords:** Meis1, pain, touch, itch, nociceptor, C-LTMR, microarray

## Abstract

Nociceptors in the dorsal root ganglia (DRG) and trigeminal ganglia (TG) are necessary for transmitting pain and itch signals. However, the molecular mechanism regulating nociceptor development remains largely unknown. This study identifies that the transcription factor *Meis1* is generally expressed in two groups of sensory neurons in the developing DRG. During prenatal and neonatal stages, approximately 2/3 of Meis1+ neurons are Runx1+ nociceptors, while 1/3 of Meis1+ neurons are NF200+ myelinated neurons. At postnatal stages, Meis1 expression in nociceptors is gradually reduced. Here, we constructed a *Meis1* conditional knockout mouse line to selectively delete *Meis1* in Nav1.8 lineage nociceptors. Microarray analyses showed that differentially expressed genes in the *Meis1* mutant DRG were enriched in pathways related to sensory perception of pain and nervous system development. In addition, *Meis1* regulates the expression of some marker genes of Nppb+ neurons and C-LTMRs. Furthermore, *Meis1* mutant mice exhibit behavioral deficits in response to light mechanical pain, static touch and chemical itch. Therefore, this study reveals that *Meis1* is required to regulate the development of nociceptors.

## Introduction

Pain and itch are unpleasant sensations triggered by harmful stimuli that evoke a withdrawal or scratching reflex to avoid potential injury. Nociceptors located in the dorsal root ganglia (DRG) and trigeminal ganglia (TG) are specialized primary sensory neurons responding to pain and itch. Single-cell transcriptome and function studies indicate that nociceptors in the DRG are divided into distinct subtypes that are recognized by the expression of a unique combination of marker genes. MAS-related GPR family member D (MrgprD)-expressing neurons mainly respond to mechanical pain and chemical itch induced by β-alanine (Cavanaugh et al., 2009; Liu et al., 2012). Two nonoverlapping subgroups of nociceptors express MAS-related GPR family member A3 (MrgprA3) and natriuretic peptide type B (Nppb). Both express a variety of itch-related molecules and transmit itch signals (Liu et al., 2009; Han et al., 2013; Mishra and Hoon, 2013; Usoskin et al., 2015; Li et al., 2016; Sharma et al., 2020). Tyrosine hydroxylase (TH) is expressed in unmyelinated C-fiber low threshold mechanoreceptors (C-LTMRs), which may be involved in touch (Li et al., 2011). Peptidergic nociceptors include diverse clusters expressing calcitonin gene-related peptide (CGRP) and neurotrophic tyrosine kinase receptor type 1 (TrkA) (Usoskin et al., 2015; Li et al., 2016; Sharma et al., 2020). All of these distinct populations are derived from initial TrkA+ precursors.

A genomic screen of transcription factor expression in the embryonic DRG shows that a limited number of transcription factors are specifically expressed in early TrkA+ neurons, suggesting that they are necessary for controlling the differentiation of nociceptors. During the embryonic period, the runt-domain transcription factor Runx1 is selectively expressed in the majority of TrkA+ nociceptors. During neonatal and postnatal development, the dynamic expression of Runx1 regulates the hierarchical development of nociceptors. Approximately 50% of nociceptors switch off Runx1 and retain TrkA expression, finally differentiating into peptidergic nociceptors. Development of this subgroup is independent of Runx1 (Chen et al., 2006). However, the remaining nociceptors require Runx1 for proper development. The persistent expression of Runx1 is necessary for the differentiation of MrgprD+ and TH+ nociceptors, while the transient expression of Runx1 is required for the development of MrgprA3+ and Nppb+ pruriceptors (Abdel Samad et al., 2010; Lou et al., 2015; Qi et al., 2017). Furthermore, the zinc finger transcription factor Zfp521 is expressed in the TH+ subgroup and segregates TH+ C-LTMR from MrgprD+ neurons (Lou et al., 2015). The homeobox transcription factor Tlx3 is broadly expressed in DRG neurons and is necessary for the development of Runx1-dependent nociceptors (Lopes et al., 2012; Huang et al., 2017).

Myeloid ectopic viral integration site (Meis) transcription factors belong to the TALE (three amino acid loop extension) homeodomain family, including Meis1, Meis2 and Meis3. During organogenesis, Meis factors play pivotal roles in regulating the development of many organs and tissues, including the skeleton, limb, eye and cerebellum (Morales and Hatten, 2006; Owa et al., 2018; Delgado et al., 2021; Dupacova et al., 2021; López-Delgado et al., 2021). Moreover, mutation of *Meis* genes is linked to tumorigenesis, such as leukemia (Imamura et al., 2002a,b) and neuroblastoma (Spieker et al., 2001; Geerts et al., 2003). We found that Meis1 was expressed in a subpopulation of DRG neurons at the embryonic stage. However, the function of Meis1 during the development of somatosensory neurons is entirely unknown. Here, we constructed a conditional *Meis1* knockout mouse line to selectively delete *Meis1* expression in nociceptors. Our studies indicated that Meis1 is required to regulate the development of nociceptors. Furthermore, behavioral studies showed that loss of *Meis1* gene impaired the behavioral response to light mechanical pain, static touch and chemical itch.

## Materials and Methods

### Animals

The generation and genotyping of *Nav1.8-Cre* transgenic mice have been described previously (Agarwal et al., 2004). The morning that vaginal plugs were observed was considered embryonic Day 0.5 (E0.5), and then the pregnant females were fed separately. Both females and males were used in the studies. All animal experiments were approved by the Animal Care and Use Committee of Beijing Institute of Biotechnology.

### Generation of *Meis1* Floxed Mice

A 12 kb genomic fragment containing *Meis1* exon 8 from the BAC clone was cloned into the retrieval plasmid carrying the Diphtheria toxin A (DTA) cassette for negative selection. The 5' *LoxP* site was inserted upstream of exon 8. A *FRT-Neo-FRT-LoxP* fragment was inserted downstream of exon 8 (Supplementary Figure 2). The *Meis1* targeting vector contained a 6.4 kb 5' recombination arm and a 3 kb 3' recombination arm. This targeting vector was electroporated into the J1 embryonic stem (ES) cell line. The G418-resistant ES clones were selected to screen for targeted ES cells with long-range PCR. Two independently targeted ES clones were injected into blastocysts derived from C57BL/6 females. Chimeric mice were mated with C57BL/6 mice to generate heterozygous mice. Germline transmission of the target allele was confirmed by PCR. Heterozygous mice were crossed with the *Flpe* deleter mouse line to remove the *neo* cassette. Mice carrying the floxed *Meis1* allele were referred to as *Meis1*^*F*^ mice. *Meis1*^*F*^ mice were mated with *Nav1.8-Cre* transgenic mice to create *Meis1*^*F*^*; Nav1.8-Cre* mice. Genotyping PCR for *Meis1*^*F*^ mice was performed with the primers 5′-GGGCTGATGGCTTTAGTGTTG-3′ and 5′- CCCGATGGCTACTACCTATTG-3′, and the wild-type allele produced a 484 bp band and the floxed allele produced a 570 bp band.

### *In situ* Hybridization and Immunofluorescence Staining

Mice were anesthetized with isoflurane and perfused with 4% paraformaldehyde in PBS (pH 7.4). DRGs were dissected and post-fixed at 4°C for 3–5 h. Tissues were dehydrated in 20% sucrose at 4°C overnight, embedded in O.C.T., and then they were stored at−80°C before use. The detailed procedures for *in situ* hybridization (ISH) and immunofluorescence (IF) staining have been described previously (Chen et al., 2006). Probes for TrkA, MrgprA3, MrgprB4, MrgprC11, MrgprD, Nppb, Sst, Cysltr2 and TH were used. The probe templates were amplified from cDNAs prepared from the mouse DRG. The probes were synthesized *in vitro* with a digoxigenin (Roche) label. The primary antibodies used for IF staining included rabbit anti-Runx1 (1:50, Abcam), rabbit anti-NF200 (1:1000, Sigma), and rabbit anti-CGRP (1:200, Peninsula) antibodies. The secondary antibodies included an Alexa Fluor 488-conjugated goat anti-rabbit antibody (1:1000, Molecular Probes), Alexa Fluor 568-conjugated goat anti-rabbit antibody (1:1000, Molecular Probes), TSA plus Fluorescein Reagent (1:1000, AKOYA), TSA plus Cy3 Reagent (1:1000, AKOYA), and Cyanine5 Amplific action Reagent (1:500, AKOYA). All primary antibodies and secondary antibodies were incubated with sections overnight at 4°C and 1.5 h at RT, respectively. Images were collected using a Zeiss confocal microscope (Zeiss LSM 800).

### RNAscope

Multilabeling RNAscope was performed according to the manufacturer's instructions (Advanced Cell Diagnostics, ACD). The following probes were used: Meis1 (436361-C1, ACD), Meis2 (436371-C2, ACD), Wfdc2 (440031-C1, ACD) and Ceacam10 (424581-C2, ACD). An RNAscope multiplex fluorescent detection reagent kit (323100, ACD) and RNA protein codetection assays (323180, ACD) were used.

### Microarray Analysis

Total RNA was extracted from the cervical, thoracic and lumbar DRGs from ~2-month-old *Meis1cko* and control mice (*n* = 3 mice per genotype) using an RNeasy Mini Kit (Qiagen) according to the manufacturer's protocol. The RNA samples were sent for microarray analysis at Beijing Kangpusen Biological Technology Company. General procedures were as follows: after quantification and qualification, the synthesis of double-stranded cDNA, *in vitro* transcription of labeled cRNA and hybridization to the GeneChip array were all performed according to standard protocols. Gene expression was analyzed using the Affymetrix mouse genome 430 v2.0 array scanned with the Affymetrix Scanner 3000. The Affymetrix GeneChip command console (version 4.0, Affymetrix) was used to analyze array images and obtain raw data. Next, Genespring software (version 12.5, Agilent Technologies) was employed to complete the basic analysis of the raw data. Differentially expressed genes were then identified based on fold changes as well as *P*-values calculated with *t*-tests. The threshold set for up- and down-regulated genes was a fold change >1.5 and a *P* < 0.05.

### Real-Time PCR

Total RNA was extracted from the cervical, thoracic and lumbar DRGs from ~2-month-old *Meis1cko* and control mice (*n* = 3 mice per genotype) using an RNeasy Mini Kit (Qiagen) according to the manufacturer's protocol. cDNA was synthesized using SuperScript II RT (Invitrogen). Meis1 primers: 5′-ATCCACTCGTTCAGGAGGAA-3′ and 5′- GTTGTCCAAGCCATCACCTT-3′. β-actin was used as control to normalize results. β-actin primers: 5′-AGATCAAGATCATTGCTCCTCCT-3′ and 5′-ACGCAGCTCAGTAACAGTCCG-3′.

### Behavior Test

Two- to three-month-old mutant and control littermates (20–30 g) of either sex were used. All behavioral tests were performed by an experimenter blinded to the genotype. The video recording was subsequently played back in slow motion to permit detailed measurements of behavioral responses directed toward the treated site. Itch and pain behavior experiments were performed as previously described (Huang et al., 2017).

For the chemical itch behavioral test, pruritic compounds were intradermally administered into the nape or cheek after mouse habituation. Behavioral responses were video recorded. The number of scratching bouts with the hindpaw and wiping bouts with the forepaw toward the injection site were counted. The following agents were used: LTD4 (5 μg/50 μl), SLIGRL-NH2 (66 μg/50 μl), chloroquine (200 μg/50 μl), 5-HT (30 μg/50 μl), Compound 48/80 (20 μg/50 μl), histamine (100 μg/10 μl), AITC (50 mM/10 μl) and capsaicin (20 μg/10 μl).

For the mechanical itch behavioral test, the napes of the mice were shaved. After habituation, the napes of the mice were stimulated 5 times with von Frey filaments (0.008–1.4 g). The numbers of scratching bouts were recorded.

For the rotarod test, each mouse was trained for 5 min at a constant speed of 4 rpm on the rotarod. The first trial started at least 1 h after training. Each mouse participated in three trials, separated by 30 min, at speeds accelerating from 4 to 40 rpm (with a 4 rpm increase every 30 s). The trial was finished when the mouse fell off the rotarod. The latency to fall off the rotarod was recorded.

For the von Frey test, the paw withdrawal threshold was determined to be ≥ 5 positive responses in a total of 10 trials with 3-min intervals after stimulation with von Frey fibers (0.008–2 g). For the pinprick test, the numbers of withdrawal responses in 10 trials with 1-min intervals were measured.

For the sticky tape assay, we placed a 9-mm diameter circular adhesive Microtube Tough-Spots on the hindpaw plantar surface and measured the latency of biting or licking to remove the tape. In addition, we placed the adhesive Microtube Tough-Spots on the nape and recorded the scratching bouts in 5 min.

For the cotton swab assay, the hindpaw plantar surface was stimulated by light stroking with a cotton swab, and the numbers of withdrawal responses in 5 trials with 1-min intervals were counted.

We placed mice on a hot plate, and the latency of hindpaw flinching/licking/jumping was measured to assess heat sensitivity. The temperature of the hot plate was set to 50, 52, and 55°C, and all animals were sequentially tested at each temperature with an interval of at least 5 min between tests. The cutoff time was set to 60 s for 50°C, 45 s for 52°C and 30 s for 55°C to avoid tissue injury.

The cold plate was set to 0°C to assess cold sensitivity. The onset of brisk forepaw flinching or hindpaw licking was assessed. For the acetone evaporation test, a drop of acetone was applied to the plantar hindpaw with a tube attached to a syringe. The time that mice spent flicking and/or licking their hindpaws after acetone application was recorded.

For the chemical pain test, capsaicin (2.5 μg/10 μl, dissolved in 7% Tween-80 in 0.9% NaCl) was administered intraplantarly. The time that mice spent licking and flinching after the capsaicin injection was measured for 3 min.

The spared nerve injury (SNI) model for neuropathic pain was established in adult control and *Meis1cko* mice (2–3 months) as described previously (Huang et al., 2017). Animals were subjected to the von Frey test at days 0–20 after lesioning. For CFA-induced inflammation, mice were briefly anesthetized with isoflurane, and 20 μl of complete Freund's adjuvant (CFA, Sigma–Aldrich) were injected into the hindpaw plantar surface. The von Frey test was performed on days 2, 3, and 6 after CFA treatment.

If the same animals were subjected to multiple behavioral tests, we firstly examined innocuous behavior, such as rotarod, von Frey and touch test, and then we performed one type of itch test 3 days later. The innocuous behavioral tests would have no carry over effect on the subsequent chemical or mechanical itch tests. A total of 253 adult mice were used for behavioral analysis in this study, including 180 males and 73 females.

### Cell Counting and Statistical Analysis

DRGs were dissected from three pairs of mutant and control mice. Each DRG was used to prepare eight sets of adjacent sections at 12 μm thickness. Each set was processed for RNAscope, ISH or IF staining. Only cells containing nuclei and showing levels of expression or staining clearly above background were counted. Representative data from experiments that were replicated biologically at least three times with similar results are shown.

For assessments of itching behaviors and acute pain, averages and SEMs were calculated for each group. The difference between the mutant and control groups was subjected to a two-tailed, unpaired Student's *t*-test, with *p* < 0.05 considered significant. For CFA-induced inflammatory and SNI-induced neuropathic pain, time-course measurements were analyzed using analysis of variance between groups (ANOVA) (within each group) and two-way repeated ANOVA (to compare groups), with *p* < 0.05 accepted as statistically significant. The results are presented as the means ± SEMs. Statistical analyses were performed using GraphPad Prism software.

## Results

### *Meis1* Expression Pattern in the DRG

**Figure 1:**
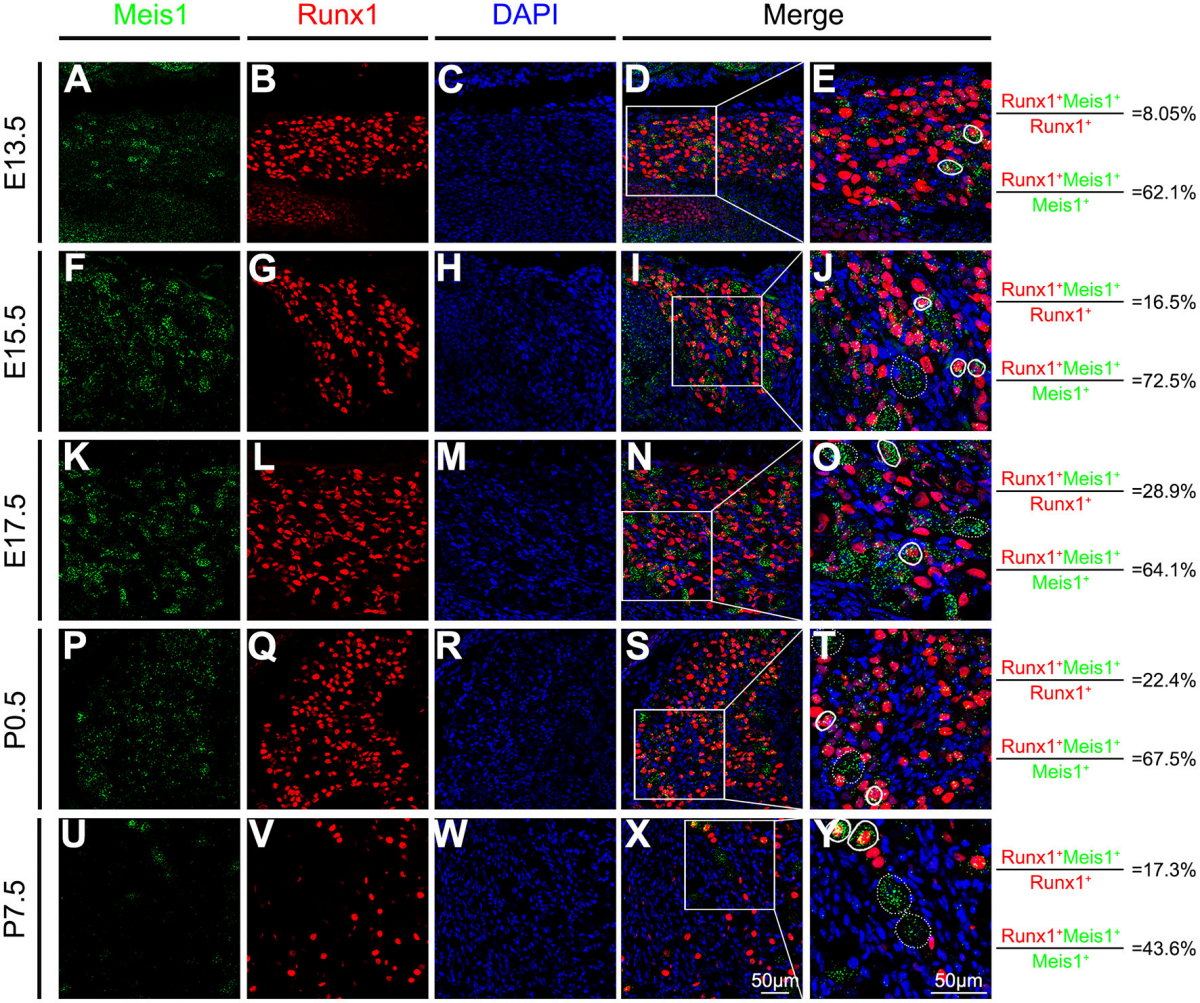
*Meis1* expression pattern in the DRG at different stages. Representative images of lumbar DRG sections costained with an antibody against the Runx1 protein (red) and *Meis1* mRNA (green) in wild-type animals at E13.5 **(A–E)**, E15.5 **(F–J)**, E17.5 **(K–O)**, P0.5 **(P–T)**, and P7.5 **(U–Y)**. Note that 8.05% (379/4711), 16.5% (367/2219), 28.9% (954/3306), 22.4% (684/3052) and 17.3% (370/2142) of Runx1+ neurons coexpressed *Meis1* at E13.5, E15.5, E17.5, P0.5 and P7.5, respectively. Additionally, 62.1% (379/610), 72.5% (367/506), 64.1% (954/1489), 67.5% (684/1013) and 43.6% (370/848) of Meis1+ neurons colocalized with Runx1 at E13.5, E15.5, E17.5, P0.5 and P7.5, respectively. **(E,J,O,T,Y)** Higher magnification views of the images shown in **(D,I,N,S,X)**. Solid lines indicate Meis1+ neurons coexpressing Runx1. *Meis1* was expressed in large neurons indicated with a dotted line. DAPI-stained cell nuclei appeared blue. Quantitative data are shown to the right of the panels. Scale bar: 50 μm.

We performed a detailed analysis of Meis1 expression in the DRG at various developmental stages to investigate its function in DRG development. TrkA+ precursor cells were first detected at embryonic day 11.5 (E11.5), whereas Meis1 was not expressed at this stage (data not shown). *Meis1* mRNA expression was first detected in the lumbar DRG at E13.5 (Figure 1A). In embryonic stages, Runx1 is specifically expressed in most, if not all, nociceptors and plays a pivotal role in controlling the differentiation of nociceptive neurons. RNAscope plus immunostaining showed that 62.1% of Meis1+ neurons coexpressed Runx1, whereas only 8.1% of Runx1+ nociceptors colocalized with Meis1 at E13.5 (Figures 1A–E). The expression level of Meis1 in the DRG increased progressively at different embryonic stages. At E15.5, 16.5% Runx1+ neurons showed *Meis1* mRNA expression (Figures 1F–J). By E17.5, *Meis1* expression reached its maximum level and was detected in 28.9% of Runx1+ nociceptors (Figures 1K–O). From E13.5 to postnatal day 0.5 (P0.5), ~2/3 of Meis1+ neurons were colabeled with Runx1. After birth, the percentage of costaining gradually decreased. At P7.5, 17.3% of Runx1+ nociceptors expressed Meis1, and 43.6% of Meis1+ neurons colocalized with Runx1 (Figures 1U–Y). Thus, *Meis1* was dynamically expressed in a subset of Runx1 lineage nociceptors during development, suggesting that Meis1 may be involved in the differentiation of nociceptor subtypes.

CGRP is a classic molecular marker of peptidergic nociceptors. We found that 20.0% of Meis1+ neurons colocalized with CGRP at P0.5 (Supplementary Figures 1A–D). Neurofilament 200 (NF200) selectively labels large A-fiber myelinated neurons. Double staining for *Meis1* and NF200 showed that 30.3% of Meis1+ neurons coexpressed NF200 at P0.5 (Supplementary Figures 1E–H), while 67.5% of Meis1+ neurons were Runx1+ nociceptors (Figures 1P–T). At P60, approximately all Meis1+ neurons coexpressed NF200 and were large in diameter. Consequently, two subgroups of Meis1-expressing neurons are identified during DRG development. One group consists of NF200+ myelinated neurons that persistently express Meis1, and the other group consists of Runx1+ nociceptors transiently expressing Meis1.

### Generation of *Meis1* Conditional Knockout Mice

**Figure 2:**
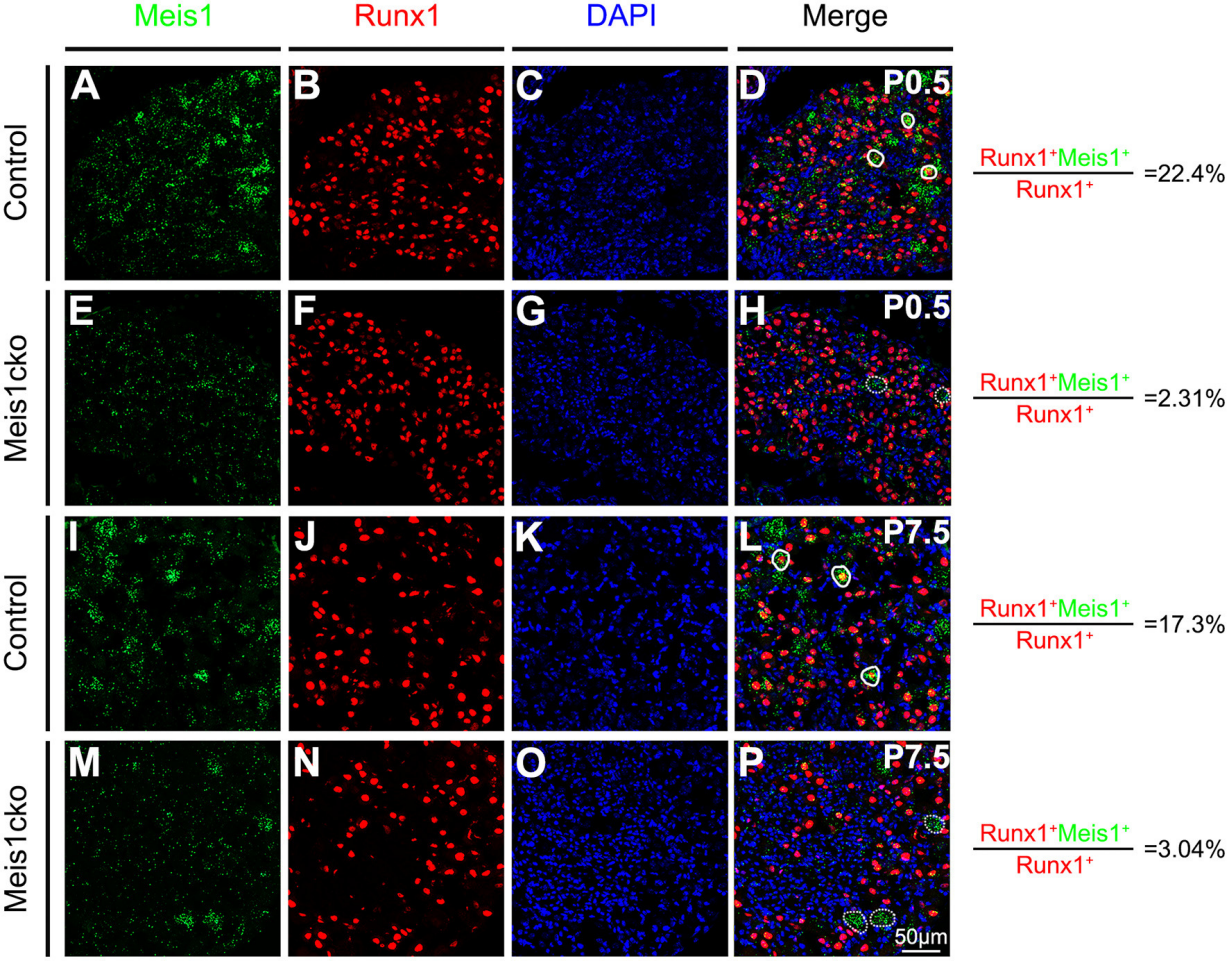
Comparing *Meis1* expression between *Meis1cko* and control DRGs. Representative images of lumbar DRG sections costained with an antibody against the Runx1 protein (red) and *Meis1* mRNA (green) in *Meis1cko* and control mice at P0.5 and P7.5. **(A–H)** 22.4% (684/3052 in control mice) and 2.31% (42/1818 in *Meis1cko* mice) of Runx1+ neurons coexpressed *Meis1* at P0.5. **(I–P)** A total of 17.3% (370/2142 in control mice) and 3.04% (33/1086 in *Meis1cko* mice) of Runx1+ neurons coexpressed *Meis1* at P7.5. Solid lines indicate Meis1+ neurons coexpressing Runx1. *Meis1* was expressed in large neurons indicated with a dotted line. DAPI-stained cell nuclei appeared blue. Quantitative data are shown to the right of the panels. Scale bar: 50 μm.

*Meis1* conventional knockout mice die before E14.5 due to extensive hemorrhaging and cardiac defects (Hisa et al., 2004) and are not suitable for determining the potential role of Meis1 in regulating DRG development at later stages. We constructed a mouse line containing a floxed *Meis1* allele (referred to as *Meis1*^*F*^), in which exon 8 encoding part of the homeodomain is flanked by two *LoxP* sites and can be removed by Cre-mediated DNA recombinase, to overcome this limitation (Supplementary Figure 2). By crossing *Meis1*^*F*^ mice with transgenic *Nav1.8-Cre* mice, in which Cre recombinase is driven from a genomic fragment derived from the *Nav1.8* promoter region and is selectively expressed in most nociceptive neurons, we generated *Meis1* conditional knockout mice (referred to as *Meis1cko*). Cre expression is first detected in *Nav1.8-Cre* mice at E16.5. *Meis1* mRNA expression in DRG was examined using the RNAscope assay to verify the efficiency of the *Meis1* gene deletion in mutant mice. *Meis1* expression in Runx1+ nociceptors was significantly decreased in *Meis1cko* mice compared to control littermates (22.4% in control mice vs. 2.31% in *Meis1cko* mice at P0.5; 17.3% in control mice vs. 3.04% in *Meis1cko* mice at P7.5, Figure 2).

### Altered Gene Expression in *Meis1cko* DRGs

We performed a microarray analysis to compare control and *Meis1cko* DRGs at the adult stage and to examine whether Meis1 contributes to the regulation of gene expression in DRG on a genome-wide scale. This analysis identified 64 upregulated and 81 downregulated genes (fold-change > 1.50, *p* < 0.05). The regulatory pathway analysis showed that differentially expressed (DE) genes were enriched in the sensory perception of pain, nervous system development, cell differentiation, regulation of apoptosis and metabolic processes (Table 1). A previous report showed that Meis1 modulates cell proliferation and differentiation by regulating metabolism (Kocabas et al., 2012). Our microarray data supported the hypothesis that Meis1 is involved in regulating the development of nociceptors.

**Table 1:** Enriched biological processes for differentially expressed genes in *Meis1cko* DRGs.

**GO biological process term**	**GENE**
Sensory perception of pain	*P2rx7; Ednrb; Grik1; Cysltr2; Galr1; Mmp2; Kif5c; Gng4; Cacna2d2*
Metabolic process	*Fbp2; Aktip; Glce; Ctsl; Kif5c; Mut; Tyrp1; Arsk*
Nervous system development	*Egr3; Sox11; Sema3b; Grik1; Nedd4; Tsc1; Ednrb; Sox17*
Regulation of apoptosis	*Prdx2; Card10; Traf4; Park2; P2rx7; Als2*
Cell Differentiation	*Prdx2; Paqr5; Wnt4; Cntn2; Meis1*

*Meis1* expression was markedly reduced in mutant DRGs but was not completely lost (Table 2). This finding was consistent with the fact that not all Meis1+ neurons were nociceptors. *Meis2* is a member of the *Meis* family and is highly homologous to *Meis1*. In developing DRGs, nearly all Meis1+ neurons coexpressed *Meis2* at different stages, and *Meis1* was only expressed in a subset of Meis2+ neurons. RNAscope identified that *Meis2* expression was similar between *Meis1* mutants and control littermates at P0.5 and P60 (Supplementary Figure 3). Consistent with this finding, microarray data showed that *Meis2* expression was normal in *Meis1cko* DRGs. At P60, *Meis1* expression was only detected in a small population of large neurons, while the expression in nociceptors was downregulated to levels not detectable (Supplementary Figure 3J).

**Table 2:** Microarray analysis showing the transcript reads of a subset of cluster-specific marker genes.

		**Transcript reads**			
**Classes**	**Gene**	**Control**	**Meis1cko**	**Foldchange**	***P*-value**
	*Meis1*	115	67	0.58	0.0214
	*Meis2*	340	317	0.93	0.7431
Nppb	*Cysltr2*	111	62	0.56	0.0099
	*Nppb*	1,661	1,490	0.90	0.5241
	*Sst*	319	275	0.86	0.5656
	*F2rl1*	203	149	0.73	0.1840
TH	*Wfdc2*	260	96	0.37	0.0007
	*Ceacam10*	374	288	0.77	0.1003
	*TH*	1,436	1,027	0.72	0.2750
	*Tafa4*	1,179	1,049	0.89	0.4705
	*Zfp521*	1,074	856	0.80	0.1233
MrgprA3	*MrgprA3*	741	874	1.18	0.3333
	*Hrh1*	41	35	0.85	0.3914

Single-cell RNA sequencing and functional studies classify the somatosensory neurons in the DRG into distinct clusters. Specific marker genes were identified in each cluster. The expression of well-known markers of mechanoreceptors and proprioceptors was similar between the two genotypes. Unexpectedly, the expression of most nociceptor-related molecules was largely unaffected in *Meis1cko* DRGs. Cysltr2, the specific receptor for LTC4 (leukotriene C4) and its downstream metabolite LTD4 (leukotriene D4), is selectively expressed in Nppb+ neurons and is related to acute and chronic itch (Solinski et al., 2019; Voisin et al., 2021). Its expression was significantly downregulated in *Meis1* mutants. Wfdc2 is specifically expressed in C-LTMRs which have been proposed to respond to light touch. Microarray data revealed an obvious decrease in *Wfdc2* expression in *Meis1cko* DRGs.

**Figure 3:**
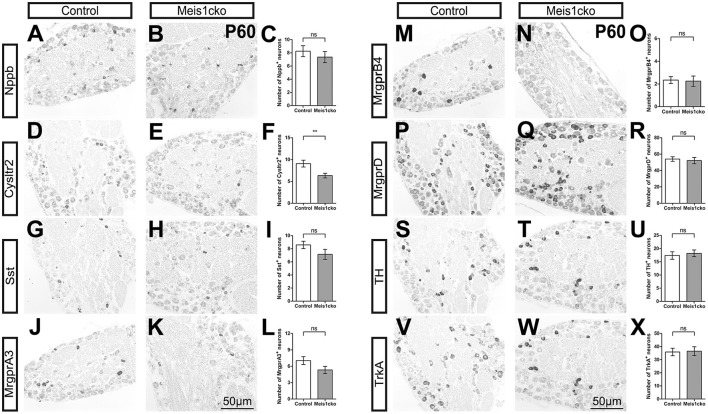
Expression of a subset of known cluster-specific markers in *Meis1cko* and control lumbar DRGs. ISH with the indicated probes on L4 or L5 lumbar DRG sections from 3 pairs of P60 *Meis1cko* and control mice. **(D–F)** *Cysltr2* expression were significantly decreased from 9.1 ± 0.8 in control DRGs to 6.3 ± 0.5 in *Meis1cko* DRGs, *p* = 0.008. **(A–C)** *Nppb*, **(G–I)** *Sst*, **(J–L)** *MrgprA3*, **(M–O)** *MrgprB4*, **(P–R)** *MrgprD*, **(S–U)** *TH*, and **(V–X)** *TrkA* expression. Error bars represent the SEM. Significant differences were determined using unpaired Student's *t*-test: **p < 0.01 ns, Not significant. Scale bar: 50 μm.

**Figure 4:**
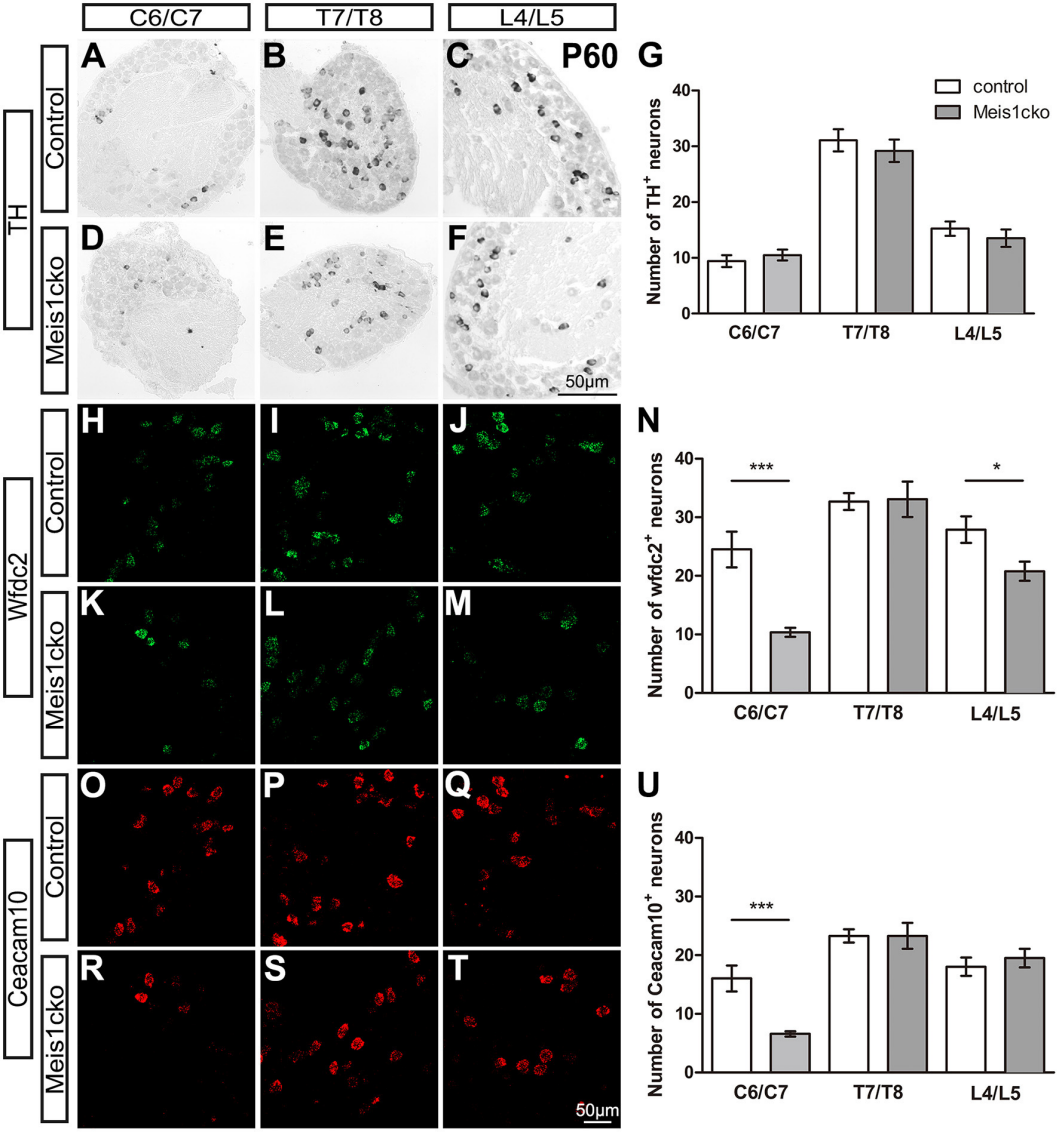
Expression of C-LTMR-specific markers in *Meis1cko* and control DRGs at different axial levels. **(A–U)** ISH and RNAscope were used to examine the expression of *TH, Wfdc2* and *Ceacam10* in cervical (C6/C7), thoracic (T7/T8) and lumbar (L4/L5) DRGs from 3 pairs of P60 *Meis1cko* and control mice. **(A–G)** *TH* expression was unchanged at different axial levels. **(H–N)** The number of Wfdc2+ neurons was significantly decreased in cervical and lumbar DRGs (in C6/C7: 24.5 ± 3.1 in control mice, 10.4 ± 0.8 in *Meis1cko* mice, *p* < 0.0001; in L4/L5: 27.9 ± 2.3 in control mice, 20.8 ± 1.6 in *Meis1cko* mice, *p* = 0.0131). **(O–U)** The number of Ceacam10+ neurons was obviously reduced in cervical DRGs (16.0 ± 2.2 in control mice, 6.6 ± 0.5 in *Meis1cko* mice, *p* < 0.0001). Error bars represent the SEM. Significant differences were determined using unpaired Student's *t*-test: *p < 0.05 and ***p < 0.001. ns, Not significant. Scale bar: 50 μm.

We selected a number of marker genes for an in-depth analysis with *in situ* hybridization and RNAscope to further verify the microarray results. We focused on the expression of cluster-specific markers. As expected, the expression of multiple nociceptor markers, including *Trpa1, Trpv1, Nppb, Sst, MrgprA3, MrgprB4, MrgprD* and *TrkA*, was almost identical between the two genotypes (Figure 3 and Supplementary Figure 4). Consistent with microarray analyses, the number of Cysltr2-positive neurons was decreased in *Meis1cko* lumbar DRGs (9.1 ± 0.8 in control mice versus 6.3 ± 0.5 in *Meis1cko* mice, n = 3 mice per group; *p* = 0.008, Figures 3D–F). According to previous studies, the expression levels of C-LTMR markers in DRGs vary at different axial levels. The relative proportion of TH+ neurons in the thoracic DRG is much higher than that in the cervical and lumbar DRG (Sharma et al., 2020). Therefore, we systematically examined the expression of *TH, Wfdc2* and *Ceacam10* in cervical, thoracic and lumbar DRGs. *TH* expression at different axial levels was normal in mutant DRGs (Figures 4A–G). *Wfdc2* expression in thoracic DRG was similar between the two genotypes, whereas the number of Wfdc2+ neurons in cervical and lumbar DRGs was significantly decreased in *Meis1cko* mice (cervical DRG: 24.5 ± 3.1 in control mice vs. 10.4 ± 0.8 in *Meiscko* mice, *p* < 0.0001; lumbar DRG: 27.9 ± 2.3 in control mice vs. 20.8 ± 1.6 in *Meis1cko* mice; *p* = 0.013. *n* = 3 mice per group, Figures 4H–N). *Ceacam10* expression in the cervical DRG was obviously reduced in mutant mice (16.0 ± 2.2 in control mice vs. 6.6 ± 0.5 in *Meiscko* mice, *n* = 3 mice per group; *p* < 0.0001, Figures 4O–U), while the expression of *Ceacam10* in thoracic and lumbar DRGs was unaffected in *Meis1cko* mice. Our findings suggested that Meis1 may play different roles in regulating the development of C-LTMR at different axial levels. We also examined the expression of a subset of markers, including MrgprD, MrgprA3, Nppb and Cysltr2, to further clarify whether Meis1 regulates the expression of other cluster-specific markers via this mechanism (Supplementary Figure 5). We observed significantly decreased *Cysltr2* expression in the thoracic DRG of *Meis1cko* mice (6.9 ± 0.6 in control mice vs. 5.0 ± 0.5 in *Meis1cko* mice, *n* = 3 mice per group; *p* = 0.014; Supplementary Figures 5P–R), yet its expression was normal in the cervical DRG. The expression of *MrgprD, MrgprA3* and *Nppb* was similar between control and *Meis1cko* DRGs at the cervical and thoracic levels (Supplementary Figure 5). Thus, Meis1 plays a role in controlling a portion of molecular features of Nppb+ neurons and C-LTMRs, including the expression of Cysltr2, Ceacam10 and Wfdc2.

We compared control and *Meis1* mutant DRGs at P0.5 and P7.5, when the expression of a few nociceptor-related markers is initiated in wild-type mice, to investigate whether Meis1 regulates the establishment of a distinct subgroup of nociceptors. At P0.5, we examined the expression of *MrgprA3, MrgprC11* and *MrgprD*. At P7.5, *Nppb, Sst*, and *Cysltr2* were first detected (Supplementary Figure 6). The mRNA expression levels of these markers were identical in control and mutant DRGs. Therefore, Meis1 is not required to induce the expression of these markers and regulates the initial differentiation of nociceptors.

### Impaired Pain Behavior in *Meis1cko* Mice

**Figure 5:**
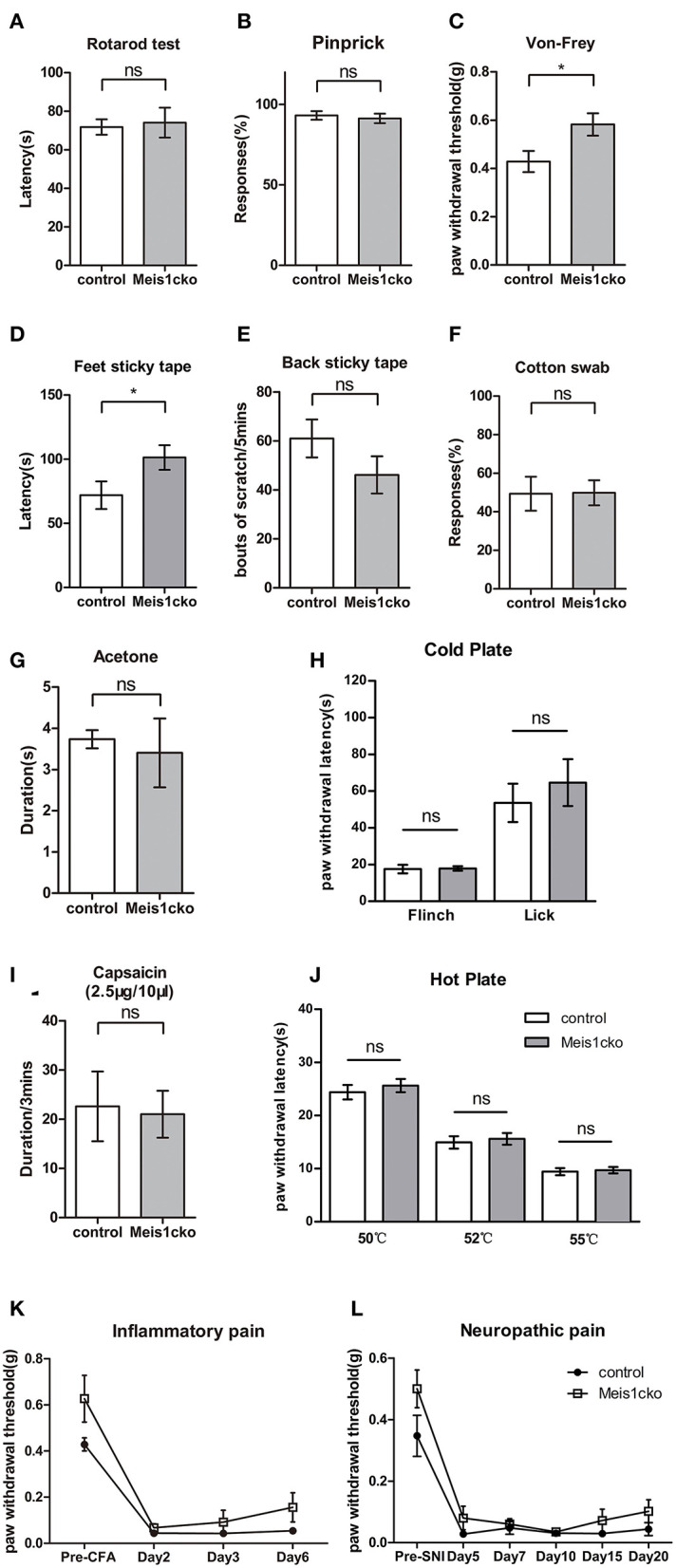
Attenuated response to static touch and light mechanical pain in *Meis1cko* mice. **(A)** The rotarod assay. Control and *Meis1cko* mice showed identical latencies to fall off the rotarod (*n* = 5 control mice, *n* = 4 *Meis1cko* mice; *p* > 0.05). **(B)** Acute mechanical pain assessed using the pinprick test. Control and *Meis1cko* mice showed no significant difference (*n* = 8 mice per group; *p* > 0.05). **(C)** Von Frey assay. *Meis1cko* mice showed a significantly higher withdrawal threshold than control littermates, and the threshold was increased from 0.43 ± 0.044 g in control mice to 0.58 ± 0.046 g in *Meis1cko* mice (*n* = 12 mice per group; *p* = 0.0187). **(D)** The latency to remove the sticky tape on the hindpaw was 72 ± 11 s for control mice and 101 ± 10 s for *Meis1cko* mice (*n* = 12 mice per group; *p* = 0.0445). **(E)** Number of scratching bouts to remove adhesive tape on nape hairy skin (61 ± 8 for control mice vs. 46 ± 8 for *Meis1cko* mice, *n* = 10 mice per group; *p* = 0.187). **(F)** Percent response to light stroking with a cotton swab (*n* = 12 mice per group; *p* > 0.05). **(G)** Cooling sensation tested by hindpaw exposure to acetone (*n* = 9 control mice, *n* = 8 *Meis1cko* mice; *p* > 0.05). **(H)** The latency of forepaw flinching or hindpaw licking on a 0°C cold plate (*n* = 10 control mice, *n* = 6 *Meis1cko* mice; *p* > 0.05). **(I)** The time that mice spent licking and flinching after the intraplantar capsaicin injection (*n* = 5 mice per group; *p* > 0.05). **(J)** Hot plate assay. No statistically significant difference was observed between control and *Meis1cko* mice at 50, 52, and 55°C (*n* = 12 mice per group, *p* > 0.05). **(K)** *Meis1cko* mice showed a normal response to CFA-induced inflammatory pain (two-way repeated ANOVA, *n* = 7 mice per group; *p* > 0.05). **(L)** *Meis1cko* mice showed a normal response to SNI-induced neuropathic pain (two-way repeated ANOVA, *n* = 6 control mice, *n* = 8 *Meis1cko* mice; *p* > 0.05). Error bars represent SEM. **(A–J)** Significant differences were determined using unpaired Student's *t*-test: *p < 0.05 ns, Not significant.

Microarray data showed that DE genes were strongly related to the pain pathway. We performed a series of acute and chronic pain assays in *Meis1cko* mice using *Meis1*^*F*/*F*^ littermates as controls to examine whether loss of *Meis1* in nociceptors affects the behavioral response to noxious stimuli. The rotarod test showed that the sensorimotor coordination of *Meis1cko* mice was similar to that of control littermates (Figure 5A). The response of mutant mice to intense mechanical pain induced by a pinprick was unimpaired (Figure 5B). In contrast, the threshold for the response to light mechanical pain delivered by von Frey filaments was increased in *Meis1cko* mice compared to control mice (0.43 ± 0.044 g in control mice vs. 0.58 ± 0.046 g in *Meis1cko* mice, *n* = 12 mice per group; *p* = 0.0187; Figure 5C). Next, we examined the response of mutant mice to innocuous touch, including static and dynamic stimulation. A sticky tape was attached to the hindpaw plantar surface to examine the static touch response of glabrous skin. The latency to remove the tape was obviously increased in mutant mice (72 ± 11 s for control mice vs. 101 ± 10 s for *Meis1cko* mice, *n* = 12 mice per group; *p* = 0.0445; Figure 5D), suggesting an impaired response to static touch. In addition, we placed sticky tape on the back of the mouse and counted the number of responses to the tape to assess the static touch response of the hairy skin. The response to this stimulus was moderately but not significantly decreased in *Meis1cko* mice (61 ± 8 in control mice vs. 46 ± 8 in *Meis1cko* mice, *n* = 10 mice per group; *p* = 0.187; Figure 5E). Furthermore, *Meis1cko* mice showed no change in response to stroking the hindpaw plantar surface with cotton swabs, indicating that a loss of Meis1 function does not affect the dynamic touch response (Figure 5F). Overall, the response of the foot to light mechanical pain and static touch was impaired in *Meis1cko* mice.

We then examined the response of mutant mice to cold and hot stimuli. Acetone evaporation on the skin rapidly reduces skin temperature to ~17°C, which is used to assess responses to innocuous cooling. Noxious cold and hot pain behaviors were examined with a cold plate assay and hot plate assay, respectively. *Meis1cko* mice exhibited no change in sensitivity to cold and heat stimuli compared to control littermates. The response to capsaicin-induced chemical pain was also not different between the two genotypes (Figures 5G–J). Both inflammatory and neuropathic pains induce chronic allodynia, and pain may be evoked by innocuous tactile stimuli. Inflammation in both control and *Meis1cko* mice was induced by injecting complete Freund's adjuvant (CFA) into the plantar pad of the hindpaw to establish inflammatory pain, as indicated by similar degrees of swelling in the hindpaws. Mechanical allodynia was not significantly different between control and *Meis1cko* mice (Figure 5K). We used the spared nerve injury (SNI) model to assess neuropathic pain. The development of mechanical allodynia following SNI remained intact in *Meis1cko* mice compared with control littermates (Figure 5L). In summary, the deletion of *Meis1* in Nav1.8 lineage nociceptors specifically impaired the response of glabrous skin to static touch and light mechanical pain but not intense mechanical pain, mechanical allodynia, or thermal and chemical pain.

### Changed Response to Chemical Itch in *Meis1* Mutants

**Figure 6:**
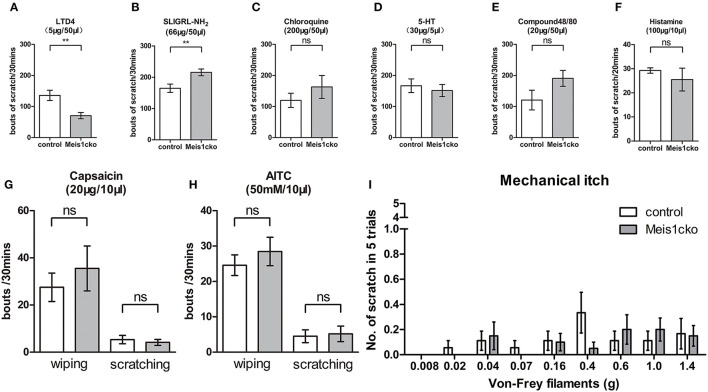
Attenuated response to LTD4-evoked itching in *Mesi1cko* mice. **(A)** The number of scratching events induced by LTD4 was significantly reduced in *Meis1cko* mice (136 ± 17 in control mice vs. 71 ± 10 in *Meis1cko* mice, *n* = 8 mice per group; *p* = 0.0046). **(B)** Scratching bouts induced by SLIGRL-NH_2_ were marginally but significantly increased in *Meis1cko* mice (165 ± 13 in control mice vs. 216 ± 11 in *Meis1cko* mice, *n* = 10 mice per group; *p* = 0.0087). **(C–F)** Control and *Meis1cko* mice showed comparable scratching behavior in response to an injection of chloroquine [*n* = 8; **(C)**], 5-HT [*n* = 9; **(D)**], or Compound 48/80 [*n* = 9; **(E)**] on the nape of mice, and histamine [*n* = 6; **(F)**] in the cheek. **(G,H)** Control and *Meis1cko* mice showed a normal response to an injection of capsaicin [*n* = 6; **(G)**] and AITC [*n* = 12; **(H)**] in the cheek. **(I)** The shaved napes were stimulated with different von Frey filaments to assess mechanical itch (*n* = 18 control mice, *n* = 20 *Meis1cko* mice). Error bars represent SEM. Significant differences were determined using unpaired Student's *t*-test: **p < 0.01 ns, Not significant.

We injected distinct pruritogens to induce chemical itch and assess whether the molecular defects in the *Meis1cko* DRG would affect itch behavior. LTD4 is an endogenous pruritogen evoking itch through the Cysltr2 receptor. Intradermal injection of LTD4 in the nape region induced a robust scratch response in control mice, while scratch bouts were obviously reduced in *Meis1cko* mice (136 ± 17 in control mice vs. 71 ± 10 in *Meis1cko* mice, *n* = 8 mice per group, *p* = 0.0046; Figure 6A), consistent with the decreased expression of Cysltr2 in mutant DRG. In contrast, a marginal but significant increase in the site-directed scratching response induced by the peptide SLIGRL-NH_2_ was observed in *Meis1cko* mice compared to control littermates (165 ± 13 in control mice vs. 216 ± 11 in *Meis1cko, n* = 10 mice per group, *p* = 0.0087; Figure 6B). The peptide SLIGRL-NH_2_ activates MrgprC11 to evoke itch, while the scratching bouts was significant decreased but not disappeared in *Mrgpr-/-* mice (Liu et al., 2011). Our microarray and *in situ* hybridization assays showed that MrgprC11 expression was largely unchanged in *Meis1cko* mice, suggesting that Meis1 may be involved in regulating the itch induced by SLIGRL-NH_2_ through another pathway. In addition, the itching response induced by the antimalarial drug chloroquine, the serotonin derivative α-Me-5-HT, Compound 48/80 and histamine was not significantly different between control and *Meis1cko* mice (Figures 6C–F). Transient receptor potential vanilloid type-1 (TRPV1) is activated by noxious heat and is also required for signaling histamine-dependent itch (Caterina et al., 1997; Shim et al., 2007). Transient receptor potential cation channel subfamily A member 1 (TRPA1) has been reported to respond to cold temperature and is required for transmitting histamine-independent itch (Bandell et al., 2004; Wilson et al., 2011). Intradermal injection of capsaicin or AITC in the cheek specifically activated TRPV1 or TRPA1 ion channels, respectively. Both scratching (itch) and wiping (pain) responses were comparable between *Meis1cko* mice and control mice (Figures 6G,H). Furthermore, mechanical itch tested by von Frey filaments was normal in mutant mice (Figure 6I).

## Discussion

### *Meis1* Is Required for the Late Differentiation of Nociceptor Subtypes

DRG neurons originate from neural crest cells that migrate from the roof plate of the fusing neural tube at the early embryonic stage. In the neurogenic phase, the neuronal determination gene *Neurogenin 2* (*Ngn2*) promotes the generation of TrkB+ mechanoreceptor precursors and TrkC+ proprioceptor precursors, while *Ngn1* is required for the formation of most TrkA+ nociceptor precursors (Ma et al., 1999). At E9.5, all sensory neurons initiate the expression of the transcription factors Brn3a and Islet1, which terminate neurogenesis and promote the induction of differentiation (Sun et al., 2008; Lanier et al., 2009). *Runx1* mRNA expression was first detected at E12.5. During embryonic stages, most TrkA+ nociceptors colocalize with Runx1, which plays a pivotal role in controlling the development of nociceptors. Conditional loss of Runx1 in Nav1.8 lineage neurons impairs the differentiation of multiple subgroups of nociceptors, including MrgprD+, MrgprA3+, Nppb+ and TH+ neurons (Abdel Samad et al., 2010; Lou et al., 2015; Qi et al., 2017). *Meis1* expression was first detected at E13.5, and the expression level gradually increased from E13.5-E17.5. The relatively late upregulation of Meis1 strongly suggests that Meis1 is not involved in the generation and early differentiation of nociceptors. Consistent with this hypothesis, the number and fate of *Meis1cko* DRG neurons were similar to those of control neurons. Our data showed that the molecular marker expression of various sensory neuron subtypes was largely unaffected in *Meis1cko* mice. Remarkably, *Meis1* mutation downregulated the expression of some late molecular markers, such as Cysltr2, Wfdc2 and Ceacam10. *Cysltr2* was first detected in the wild-type DRG at P7.5 (Supplementary Figure 4), while *Ceacam10* and *Wfdc2* were first detected in the wild-type DRG at P9.5 (data not shown). Furthermore, *Meis1* mutation impairs the behavioral response to static touch, light mechanical pain and chemical itch. Consequently, these data suggest that Meis1 may regulate the late differentiation and maturation of sensory neuron subtypes.

The Runx1-dependent transcription factor Zfp521 was detected in DRGs after E13.5 and was mainly expressed in TH+ C-LTMRs at P30. During development, Zfp521 is required to control the molecular identities of C-LTMRs and segregate TH+ neurons from MrgprD+ neurons (Lou et al., 2015). Meis1 is also involved in regulating the development of TH+ C-LTMRs, including the expression of Wfdc2 and Ceacam10. Nevertheless, TH and TAFA4 expression, which were markedly downregulated in both *Runx1cko* and *Zfp521cko* mice (Lou et al., 2013, 2015), were largely unaffected in *Meis1cko* mice. Therefore, the mechanism by which Meis1 controls the development of C-LTMRs is different from that of Runx1 and Zfp521.

Alignment of the DNA sequence in *Meis* genes shows high homology between *Meis1* and *Meis2* genes. These genes encode highly similar proteins. Both *Meis1* and *Meis2* constitutive knockout mice are embryonically lethal due to extensive hemorrhaging and cardiovascular defects. During eye development, *Meis1* and *Meis2* functionally compensate for each other, indicating a redundant relationship. In addition, deletion of both genes results in a more serious deficiency in the limb and the vertebrate axial skeleton compared to single mutation (Delgado et al., 2021; Dupacova et al., 2021; López-Delgado et al., 2021). In the developing DRG, almost all Meis1-expressing neurons coexpress Meis2, and Meis1 is expressed in a subset of Meis2+ neurons. Our findings prompted us to hypothesize that the lack of severe phenotypic defects in the *Meis1* single mutant is likely due to functional compensation by Meis2, which maintains the development of nociceptors. In the future, the construction of mice with a double conditional deletion of *Meis1* and *Meis2* in nociceptors would be helpful to clarify Meis function during nociceptor development.

Whether the behavioral deficits in response to static touch and light mechanical pain are due to the impaired C-LTMR differentiation and/or the changed expression of several genes related to pain pathway remains unclear and will need to be clarified in future studies. Our findings further reveal the molecular mechanism of nociceptor development. Moreover, our results showed that *Meis1* mutant mice exhibited impaired mechanical pain and chemical itch, and some of Meis1-dependent genes expressed ion channels or receptors related to pain and itch, such as *Cacna2d2, P2rx7* and *Cysltr2*. Thus, targeting these proteins may be promising for developing the novel therapeutic approach for pain and itch management.

### Meis1 Regulates the Development of C-LTMRs Along the Anterior-Posterior Axis

During skeleton and limb development, Meis proteins collaborate with position factors to establish anterior-posterior (AP) and proximo-distal (PD) patterning, which provide critical spatial information for body planning and the progressive development of distinct body segments. The sequential expression and activation of Hox during axial elongation is generally considered the translation of temporal information into spatial information (Deschamps and Duboule, 2017). ChIP-seq analysis shows that genomic Hox-binding sites highly overlap with Meis-binding sites, suggesting that Hox proteins bind to DNA by directly or indirectly interacting with Meis proteins. On the other hand, Meis-binding sites are also detected in the vicinity of *Hox* genes, suggesting that Meis proteins may regulate *Hox* gene transcription. Temporally and spatially dynamic Meis expression in limb buds controls the timing of activation and position of the HoxA complex, which are necessary for establishing limb PD patterning and proximal segments (Delgado et al., 2020). Moreover, Meis proteins are required to regulate the expression and activation of several patterning genes, including *Hoxd9, Hoxd13* and *Hand2*, which establish forelimb AP patterning (Delgado et al., 2021). Deletion of both *Meis1* and *Meis2* leads to limb agenesis. In addition, skeleton AP patterning is mediated by the Hox-Meis-Pbx complex (López-Delgado et al., 2021). Loss of Meis1 and Meis2 function produces axial skeletal defects in the cervical and thoracic regions, including anterior homeotic transformation of the cervical vertebrate and rib mispatterning. Taken together, Meis proteins play different roles at distinct axial levels. The development of the spinal cord and DRG is also controlled by AP patterning. The development stage of the anterior spinal cord segments occurs earlier than that of the posterior part. The relative proportions of various DRG neuron subtypes differ in distinct segments along the AP axis, such as C-LTMRs and proprioceptors (Sharma et al., 2020). In the present study, the expression of Wfdc2 and Ceacam10 in the *Meis1cko* cervical DRG was markedly decreased compared to that in the control DRG, yet their expression in the thoracic DRG was largely unchanged in *Meis1* mutants, suggesting that Meis1 may regulate the development of C-LTMRs along the AP axis. Our microarray data show that the expression levels of *Hox* and *Pbx* genes are similar between *Meis1cko* and control DRGs, indicating that Meis1 is not required for regulating the expression of these position factors. Further study is required to identify the molecular mechanism by which Meis proteins regulate the development of the DRG along the AP axis.

## Data Availability Statement

The datasets presented in this study can be found in online repositories. The names of the repository/repositories and accession number(s) can be found at: https://www.ncbi.nlm.nih.gov/geo/, GSE199051.

## Ethics Statement

The animal study was reviewed and approved by Animal Care and Use Committee of Beijing Institute of Biotechnology.

## Author Contributions

ZC performed the research, analyzed the data, and wrote the manuscript. CH, FL, XJ, and YH performed the research. CC analyzed data and supervised the research. ZL designed, supervised the research, analyzed the data, and wrote the manuscript. All authors contributed to the article and approved the submitted version.

## Funding

This study was supported by National Natural Science Foundation of China (Grant 31371102).

## Conflict of Interest

The authors declare that the research was conducted in the absence of any commercial or financial relationships that could be construed as a potential conflict of interest.

## Publisher's Note

All claims expressed in this article are solely those of the authors and do not necessarily represent those of their affiliated organizations, or those of the publisher, the editors and the reviewers. Any product that may be evaluated in this article, or claim that may be made by its manufacturer, is not guaranteed or endorsed by the publisher.

## Supplementary Material

Supplementary Figure 1 *Meis1* expression pattern in the DRG. Representative images of lumbar DRG sections costained with the markers CGRP (green), NF200 (green) and *Meis1* mRNA (red) in wild-type mice at P0.5. **(A-D)** A total of 20.0% (144/719) of Meis1+ neurons colocalized with CGRP. **(E-H)** A total of 30.3% (184/607) of Meis1+ neurons coexpressed NF200. DAPI-stained cell nuclei appeared blue. Quantitative data are shown to the right of the panels. Scale bar: 50 μm.

Supplementary Figure 2 Diagram showing the construction of a *Meis1* conditional knockout mouse line. **(A)** A *neo* cassette flanked by two *Frt* sites and *exon 8* (E8) flanked by *LoxP* sites were designed. By crossing *Flpe* and *Nav1.8-cre* transgenic mice, *Meis1* conditional knockout mice were generated. **(B)** RT-PCR showed that *Meis1* mRNA level in *Meis1cko* DRGs was decreased to approximately 70% of controls (*n* = 3). ^*^*p* < 0.05.

Supplementary Figure 3 Meis1+ neurons coexpressed Meis2 at different stages. Representative images of lumbar DRG sections costained with the *Meis1* mRNA (green) and *Meis2* mRNA (pink) in *Meis1cko* and control mice at different stages. Nearly all Meis1+ neurons coexpressed Meis2. DAPI-stained cell nuclei appeared blue. Scale bar: 50 μm.

Supplementary Figure 4 Expression of *Trpa1* and *Trpv1* was similar between control and *Meis1cko* DRG. **(A–C)** *Trpa1* expression on T13 DRG (*n* = 3), **(D–F)** *Trpv1* expression on L4 DRG (*n* = 3). Scale bar: 50 μm.

Supplementary Figure 5 Distinct cluster marker expression patterns at different axial levels. **(A–X)** ISH with the indicated probe on sections through C6/C7, T7/T8 or L4/L5 DRGs from 3 pairs of P60 *Meis1cko* and control mice. The expression of *MrgprD* **(A–F)**, *MrgprA3* **(G–L)** and *Nppb* **(S–X)** was not significantly altered in cervical and thoracic DRGs. **(M–R)** The expression of *Cysltr2* in the thoracic DRG was significantly decreased from 6.9±0.6 in control mice to 5.0 ± 0.5 in *Meis1cko* mice (*n* = 3 mice per group, *p* = 0.014), but a significant change was not observed in the cervical DRG. Error bars represent the SEM. Significant differences were determined using unpaired Student's t test: ^*^*p* < 0.05, ns, Not significant. Scale bar: 50 μm.

Supplementary Figure 6 Expression of a subset of known cluster-specific markers in *Meis1cko* and control DRGs at P0.5 and P7.5. ISH with the indicated probe on lumbar DRG sections from 3 pairs of *Meis1cko* and control mice at P0.5 **(A–I)** and P7.5 **(J–R)**. Error bars represent the SEM. Significant differences were determined using unpaired Student's t test: ns, Not significant. Scale bar: 50 μm.
