# Supplementary figures and images for: Meis1 Regulates Nociceptor Development and Behavioral Response to Tactile Stimuli
Source: Front Mol Neurosci. 2022 Jul 6;15:901466. doi: 10.3389/fnmol.2022.901466 (PMC9301487; doi:10.3389/fnmol.2022.901466)

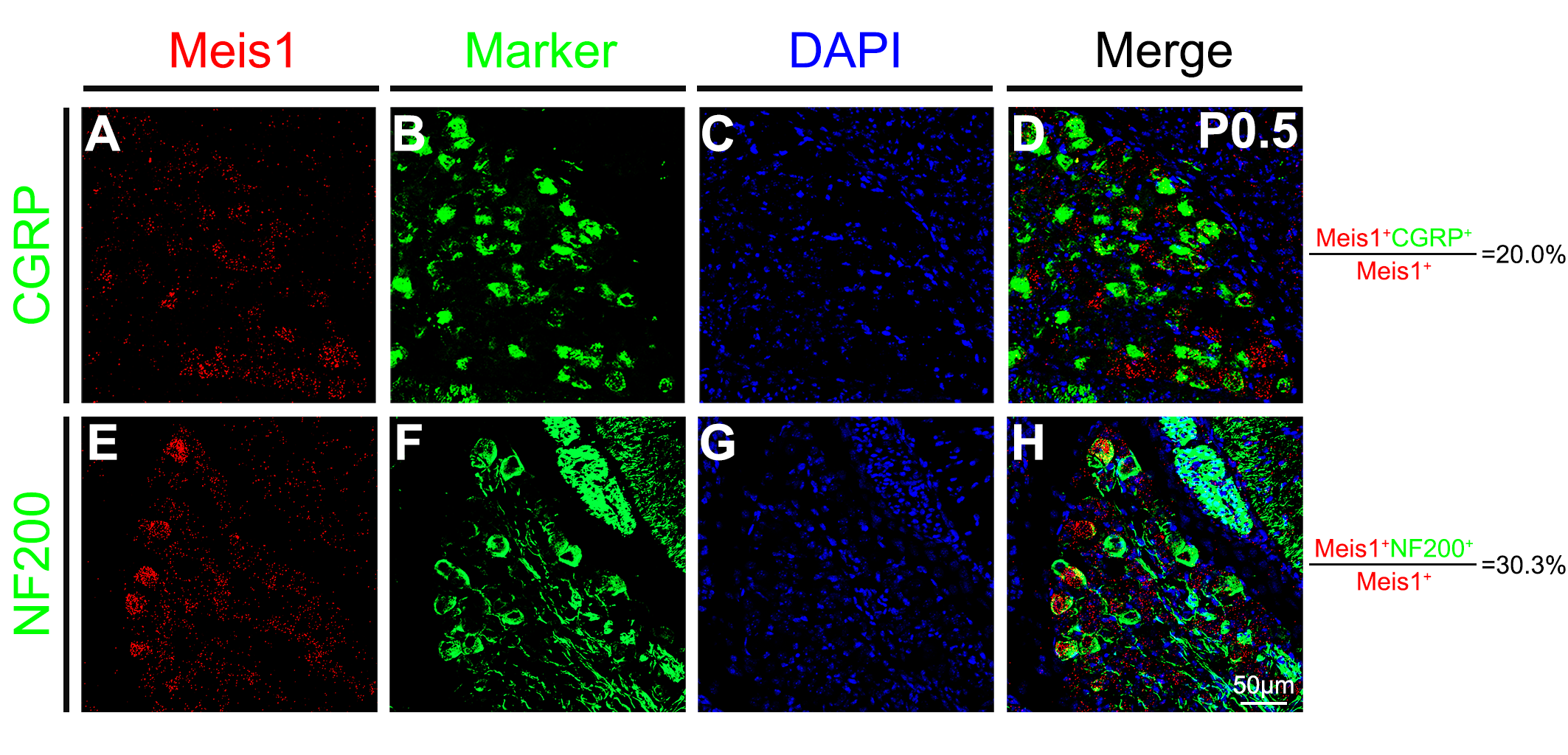

Supplement: Supplementary Figure 1 — Meis1 expression pattern in the DRG. Representative images of lumbar DRG sections costained with the markers CGRP (green), NF200 (green) and Meis1 mRNA (red) in wild-type mice at P0.5. (A-D) A total of 20.0% (144/719) of Meis1+ neurons colocalized with CGRP. (E-H) A total of 30.3% (184/607) of Meis1+ neurons coexpressed NF200. DAPI-stained cell nuclei appeared blue. Quantitative data are shown to the right of the panels. Scale bar: 50 μm. [file Image_1.TIF]

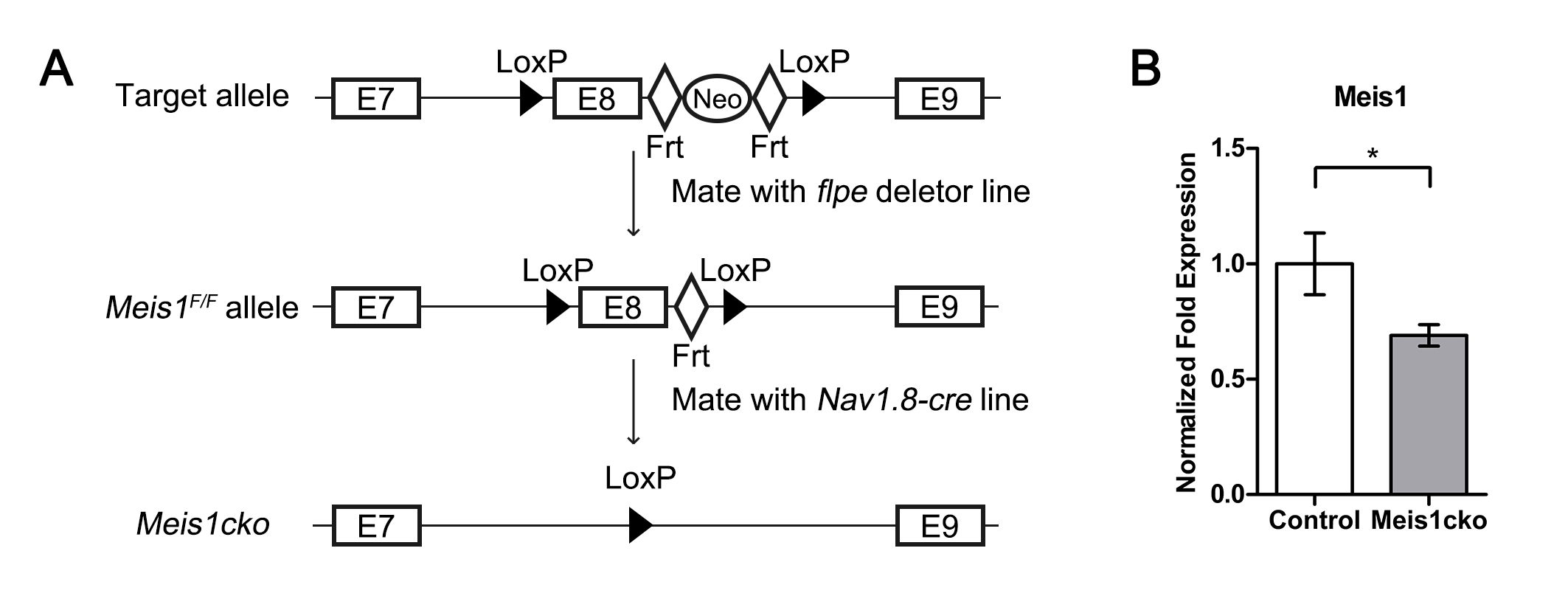

Supplement: Supplementary Figure 2 — Diagram showing the construction of a Meis1 conditional knockout mouse line. (A) A neo cassette flanked by two Frt sites and exon 8 (E8) flanked by LoxP sites were designed. By crossing Flpe and Nav1.8-cre transgenic mice, Meis1 conditional knockout mice were generated. (B) RT-PCR showed that Meis1 mRNA level in Meis1cko DRGs was decreased to approximately 70% of controls (n = 3). *p < 0.05. [file Image_2.TIF]

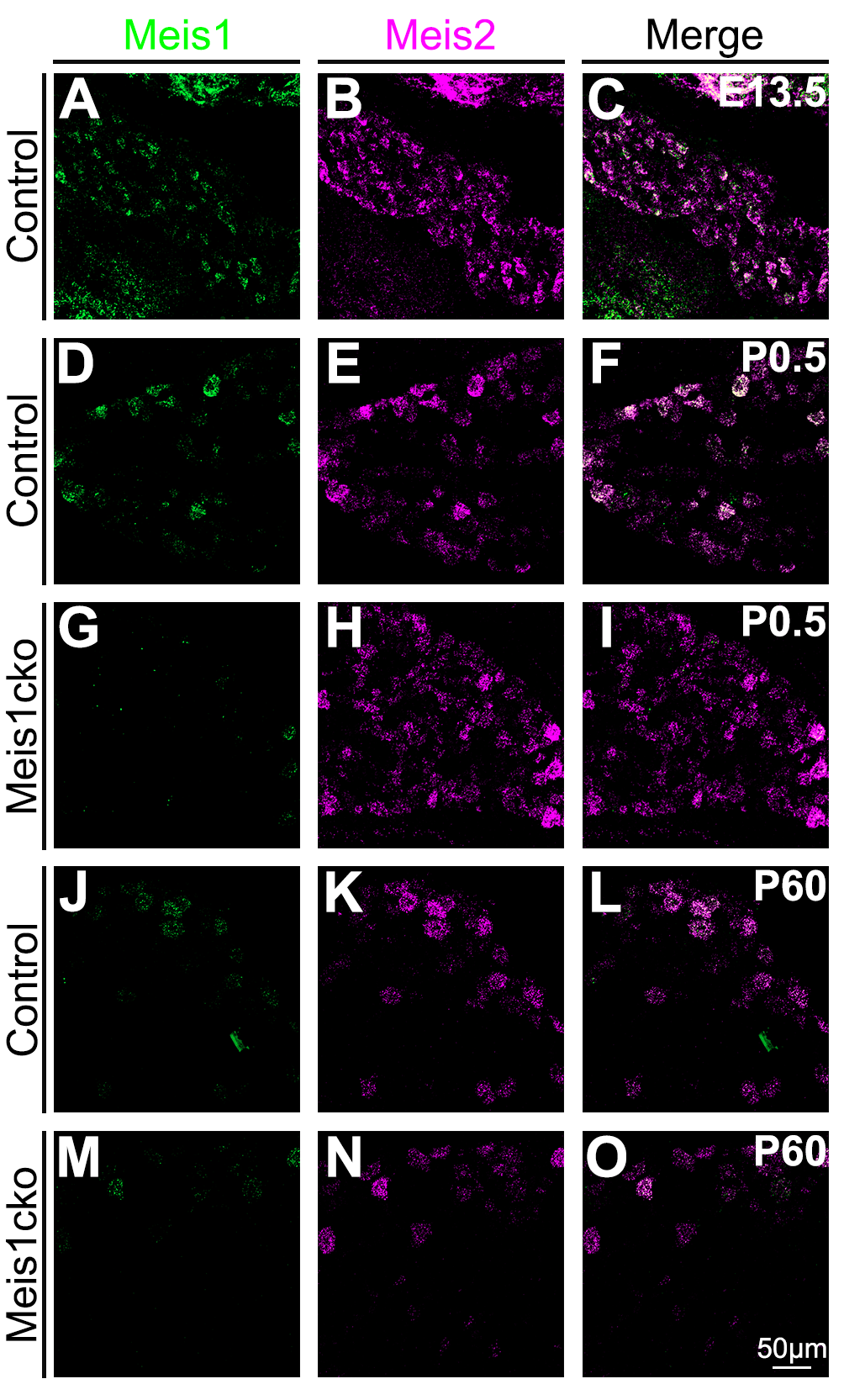

Supplement: Supplementary Figure 3 — Meis1+ neurons coexpressed Meis2 at different stages. Representative images of lumbar DRG sections costained with the Meis1 mRNA (green) and Meis2 mRNA (pink) in Meis1cko and control mice at different stages. Nearly all Meis1+ neurons coexpressed Meis2. DAPI-stained cell nuclei appeared blue. Scale bar: 50 μm. [file Image_3.TIF]

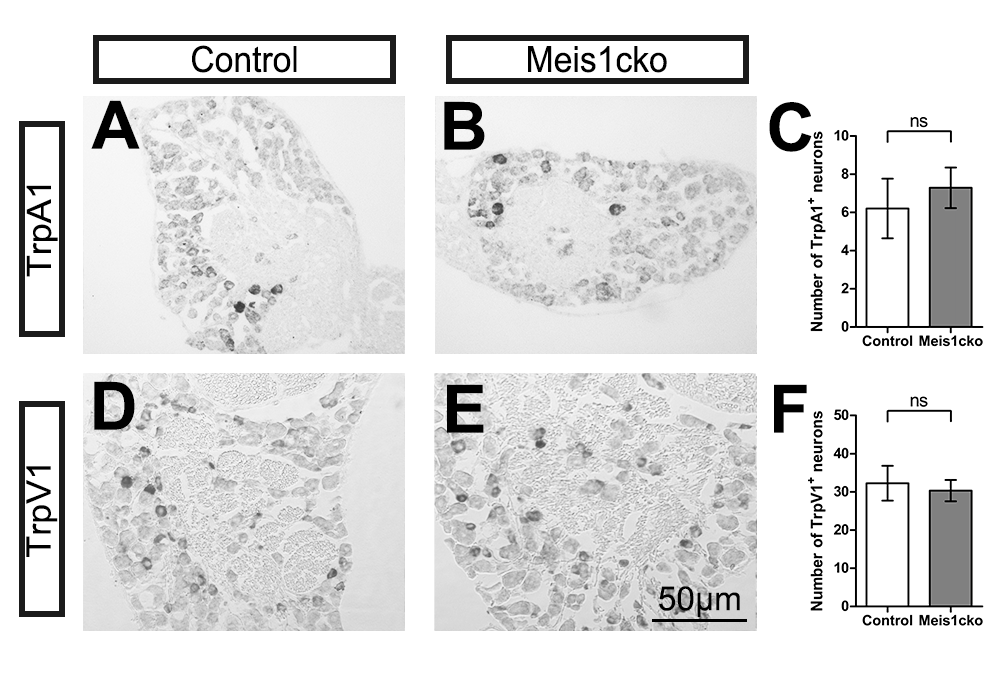

Supplement: Supplementary Figure 4 — Expression of Trpa1 and Trpv1 was similar between control and Meis1cko DRG. (A–C) Trpa1 expression on T13 DRG (n = 3), (D–F) Trpv1 expression on L4 DRG (n = 3). Scale bar: 50 μm. [file Image_4.TIF]

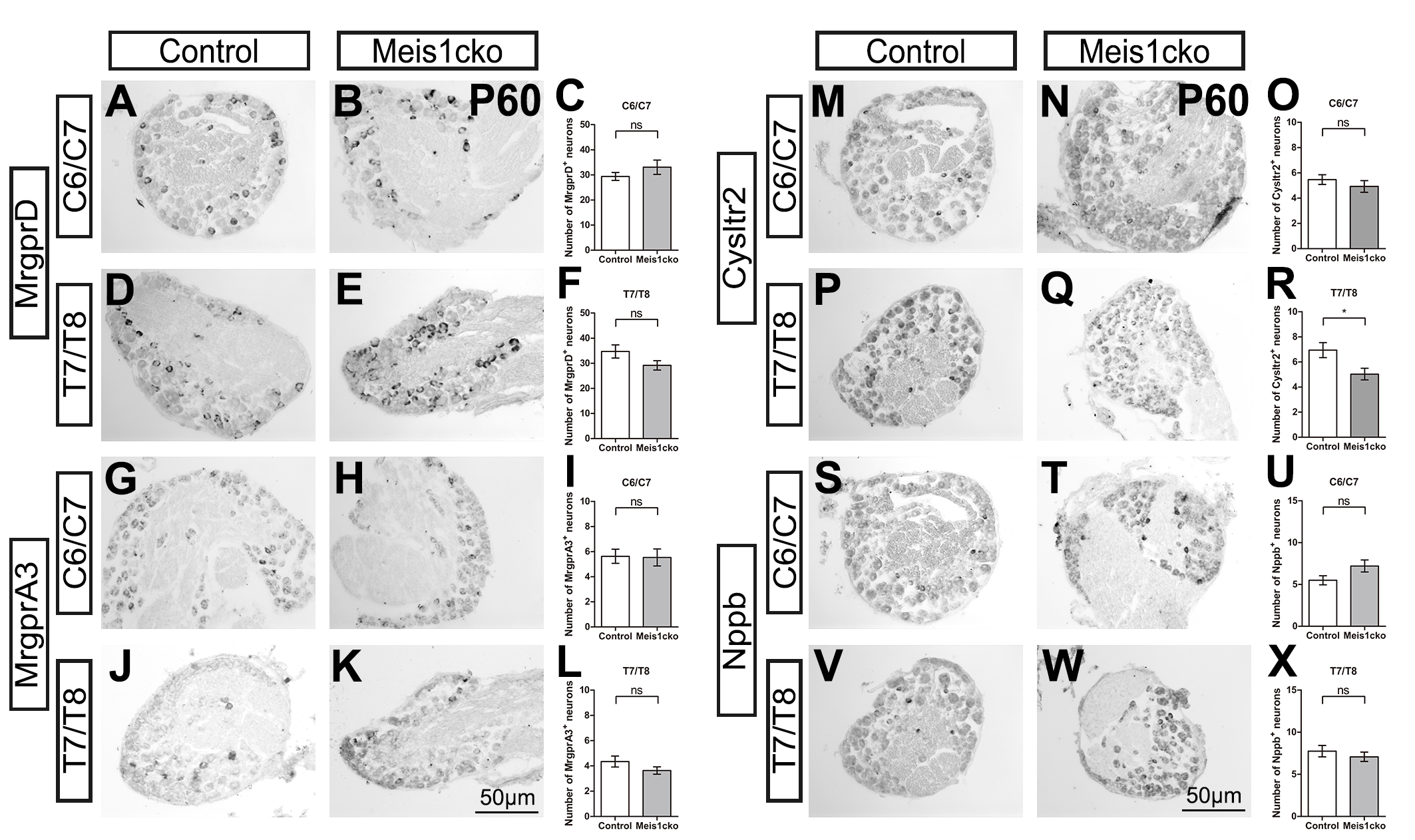

Supplement: Supplementary Figure 5 — Distinct cluster marker expression patterns at different axial levels. (A–X) ISH with the indicated probe on sections through C6/C7, T7/T8 or L4/L5 DRGs from 3 pairs of P60 Meis1cko and control mice. The expression of MrgprD (A–F), MrgprA3 (G–L) and Nppb (S–X) was not significantly altered in cervical and thoracic DRGs. (M–R) The expression of Cysltr2 in the thoracic DRG was significantly decreased from 6.9±0.6 in control mice to 5.0 ± 0.5 in Meis1cko mice (n = 3 mice per group, p = 0.014), but a significant change was not observed in the cervical DRG. Error bars represent the SEM. Significant differences were determined using unpaired Student's t test: *p < 0.05, ns, Not significant. Scale bar: 50 μm. [file Image_5.TIF]

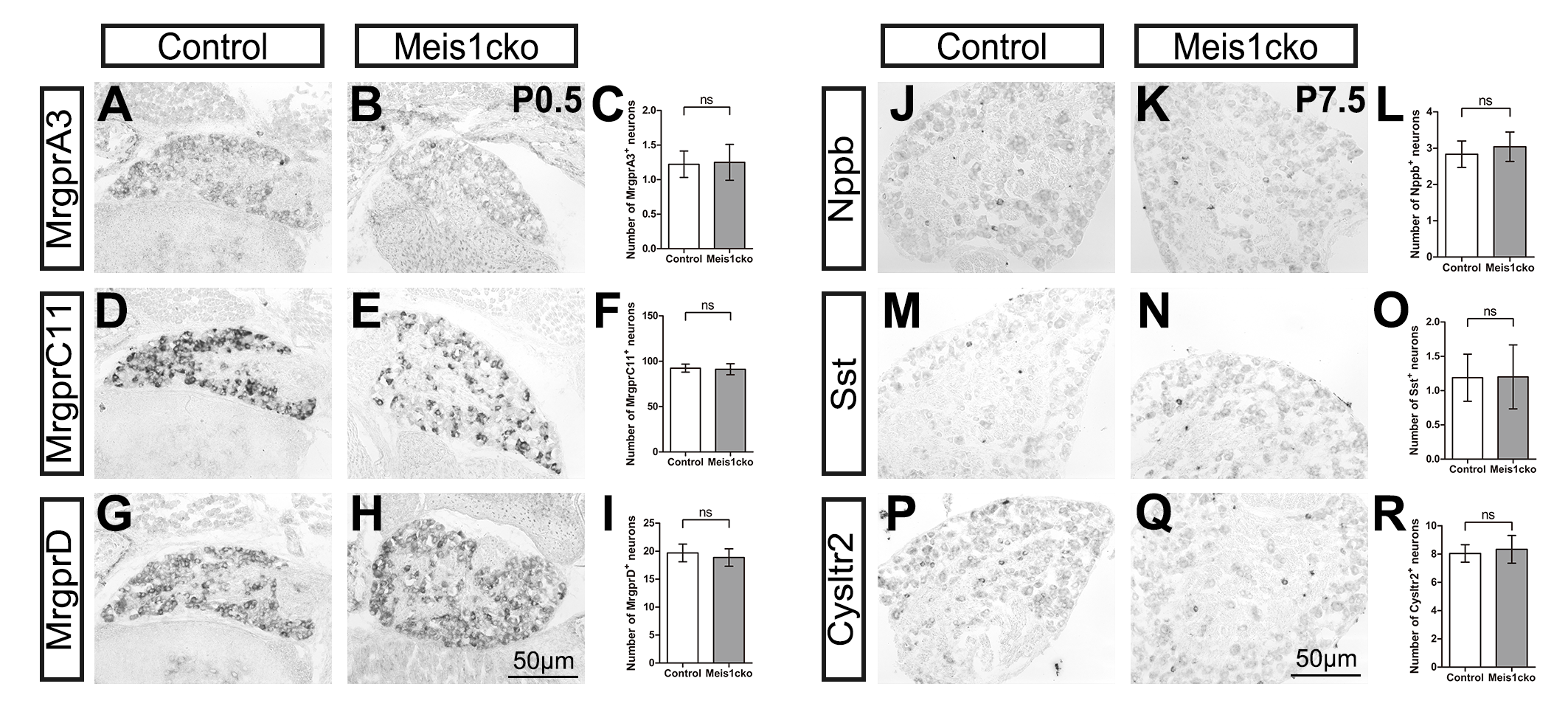

Supplement: Supplementary Figure 6 — Expression of a subset of known cluster-specific markers in Meis1cko and control DRGs at P0.5 and P7.5. ISH with the indicated probe on lumbar DRG sections from 3 pairs of Meis1cko and control mice at P0.5 (A–I) and P7.5 (J–R). Error bars represent the SEM. Significant differences were determined using unpaired Student's t test: ns, Not significant. Scale bar: 50 μm. [file Image_6.TIF]
